# Consumption Threshold at Which Virtue Products Become Vice Products: The Case of Beer

**DOI:** 10.3390/foods10081688

**Published:** 2021-07-22

**Authors:** Enar Ruiz-Conde, Francisco Mas-Ruiz, Josefa Parreño-Selva

**Affiliations:** Department of Marketing, Faculty of Economics, University of Alicante, P.O. Box 99, E-03080 Alicante, Spain; francisco.mas@ua.es (F.M.-R.); pepi@ua.es (J.P.-S.)

**Keywords:** consumption threshold, virtue and vice products, self-control, reverse self-control problem

## Abstract

Relative vices and virtues have traditionally been defined according to time-inconsistent preferences. Vice products exchange small immediate rewards (e.g., pleasure) for larger delayed costs (e.g., health), while virtue products exchange small immediate costs for larger delayed rewards. This definition can be criticized because there is evidence that small amounts of beer (or chocolate) convey a long-term health benefit, whereas large quantities impose a delayed cost. Thus, we assume that virtue products can become vice products when consumption is above a certain threshold. Survey data identifies alcoholic beer as a product that gives immediate rewards and does not impose a delayed cost. Our analysis reveals a consumption threshold that supports our assumptions.

## 1. Introduction

One widely accepted definition of relative vices (see [1,2,3,4,5,6,7,8,9]) considers them as products that provide an immediately gratifying experience (e.g., the good taste of regular-fat cream cheese), but have negative long-term outcomes (e.g., future health problems). Conversely, relative virtues are seen as less gratifying and less appealing in the short term (e.g., inferior flavour of light cream cheese) but have fewer negative long-term consequences and are a more prudent choice. However, people may consume some products with an immediate reward while not regarding them as imposing a delayed, non-pecuniary cost. For example, beer is perceived in Spain as a natural product of low alcoholic strength that forms part of a healthy Mediterranean diet and is associated with moderate consumption at social gatherings [10]. There is even evidence that small amounts of beer (or, to cite another example, chocolate) convey long-term health benefits, whereas large quantities impose a delayed cost (e.g., [11,12]). In this sense, the relevant question would be whether there is a certain consumption threshold at which a virtue turns into a vice.

The existence of a consumption threshold has been suggested by authors such as [13]; they determine that establishing the healthiness of a product is notoriously difficult because it is not an inherent quality of food; because it depends on the conditions in which the food is eaten, and also on the quantity eaten. Specifically, two areas have been researched. Firstly, how to market products so that consumers make better food choices (see [14,15]) and secondly, how to help consumers make changes in their environment to help control their food consumption (see [16]). Both lines offer advice not only to the food industry but also to consumers because, despite the presence of the widely held belief that ‘unhealthy = tasty’ [17], consumers can make food choices that are both healthy and enjoyable [18,19]. The possibility to control the effect of this ‘unhealthy = tasty’ perception (and its potential to generate negative health consequences) should include the control of the volume of unhealthy but tasty food that is eaten. If consumers believe that unhealthy food is tastier, then naturally that would generate a strong desire to eat food that is relatively unhealthy. One possible solution to this problem would be for both marketers and consumers to take steps to ensure that if these foods are going to be consumed then it should be in reasonable quantities [13]. Thus, a relevant research gap is to study whether there is a certain consumption threshold at which a virtue turns into a vice.

The analysis of a consumption threshold at which a virtue turns into a vice also has important practical implications for marketers, such as knowing whether to distinguish vice and virtue products in price promotions (see [1]); or whether to focus on price promotions of regular products, where consumption above a certain threshold turns a virtue into a vice. At the same time, there is a debate in many firms around whether to continue offering unhealthy products while announcing plans to improve nutrition (in this debate there is a lack of agreement on the definition of ‘healthy’ and ‘unhealthy’ food). This debate could benefit from the consumption threshold approach because it would incentivize the use of nutritional labelling to indicate the amount of a nutrient relative to the recommended amount (see [20]).

We contribute to the development of knowledge on this topic of consumption threshold. The aim of our study is to measure whether there is a delayed cost to consumption. First, we examine whether there are specific ‘vice’ products that do not impose a delayed non-pecuniary cost. Second, we analyse whether consuming large quantities of the product imposes a delayed cost. Survey data from students of a university in Spain identify alcoholic beer as a product that has an immediate reward and does not impose a delayed cost. In addition, our main results reveal a threshold at which a virtue turns into a vice, as described in our assumption.

## 2. Consumption Patterns for Products That Do Not Induce Time-Inconsistent Preferences

The distinction between relative vice and virtue products revolves around consumers’ order of preference when evaluating immediate or delayed consequences of consumption [1]: for example, if long term health benefits are ignored many smokers prefer regular cigarettes (relative vice) as opposed to light (relative virtue) because they prefer the flavour. However, if short term flavour preferences are ignored and the long term health benefits of smoking are considered then these same smokers prefer light over regular cigarettes. This ordering of preferences could lead to dynamically inconsistent choices, when the immediate and delayed consequences depend on the time lag between purchase and consumption [1,21]: for example, the choice made by a dieter, when offered either chocolate cake or fresh fruit as a dessert could vary depending on whether the choice is made before the meal (the moment of purchase in the shop) or during the meal (at home when both the cake and the fruit are available immediately).

In addition, this definition of relative vices and virtues according to time-inconsistent preferences is based on theoretical assumptions of self-control and reverse self-control. Consumers can show time-inconsistent preferences between relative vices and virtues, and according to the assumption of potential self-control (see [1]), they are likely to show control over the purchase of vice products because people are easily tempted by unhealthy but tasty products. The self-imposed constraint of rationing the quantity bought gives people self-control as it limits stock and, therefore, opportunities for consumption. However, the traditional self-control model has been criticized for its short-sightedness and present-biased preferences (‘myopia’) (see [22,23]) with reference to the moment of choice between immediate temptations and long-term gains. This model has been challenged [24] by the concept of hyperopia, because the consumer also has a reverse self-control problem, characterized by excessive farsightedness and future-biased preferences [25].

Excessive control and farsightedness (hyperopia) can have negative long-term consequences in the sense that over time, the choices of virtue over vice evoke an increase in regret [22]. This logic is based on the idea that over time, guilty feelings from vice consumption diminish and the sense of loss of the pleasures of life intensifies [23]. This long-term regret, which is associated with excessive control, relaxes the power of self-control and motivates consumers to buy vice products.

Theoretical assumptions of self-control and reverse self-control derive from a definition of relative vice and virtue products according to time-inconsistent preferences. However, this can be criticized, since consumers may perceive products as giving an immediate reward and having no delayed cost (i.e., products that do not induce time-inconsistent preferences—see Section 3.1). This would suggest no self-control over consumption nor reverse self-control, meaning that the quantities purchased are not dependent on the vice or virtue nature of the product. Alternatively, we wish to obtain a definition based on the relevant question of whether there is a certain consumption threshold at which a virtue turns into a vice.

Health sciences (see [20,26,27]) and consumer research (see [28,29]) basically suggest that there is a fine line between healthy and risky consumption of a vice product. Ample empirical evidence shows that the consumption of small quantities of alcohol is healthy [30,31,32]. Similarly, moderate consumption of fat and carbohydrates has been found to have beneficial health effects (see [13]). A hamburger can be ‘energy dense’ because it is high in calories, but nutritionists consider it to have some nutritional value [33]. Theory on how individuals consume varying food sizes, package sizes, and portion sizes provides some direction regarding the idea that consumers have come to expect large food portions (see [34,35,36]), in part due to perceived lower food costs [37,38]. However, seeing small amounts of food in small packages could reinforce the idea that this configuration is the diet version [29]. In fact, there are numerous examples of smaller portions in smaller packages as representations of diet foods [28], such as 100-calorie snack packages.

In brief, we extend this approach by assuming that consumers can perceive a consumption threshold at which point there would be a delayed cost; thus, they see their consumption as above or below the threshold at which virtue becomes vice.

## 3. Research Design

Analysis of the objectives of our study required a specific research design to examine the following aspects: (i) whether there are specific ‘vice’ products that do not impose a delayed non-pecuniary cost; that is, they do not induce time-inconsistent preferences (see Section 3.1); (ii) whether there is a consumption threshold at which virtue products turn into vice products (see Section 3.2); (iii) the lack of consumption self-control, which was measured using an experiment that tested the effect of price promotions on quantity purchases of vice and virtue products (see Section 3.3); and (iv) the lack of reverse self-control through an experiment that tested the effect of passage of time on anticipated regret over vice and virtue product purchases (see Section 3.4).

The study’s sample comprised 176 business and administration students (340 students received the questionnaire included in the Appendix A, 52% of whom replied) of a university in Spain. Similar sized samples are used in other experiments to analyse vice and virtue products. For example, [1] used a sample of 136 MBA students to identify vice and virtue products; and [22] employed a sample of 132 university students to establish the effect of self-control regret when faced with a choice between fruit salad and chocolate cake.

### 3.1. Identification of Specific ‘Vice’ Products That Do Not Impose a Delayed Non-Pecuniary Cost

To identify whether there are specific ‘vice’ products with no delayed costs, we used the procedure of [1], which is based on a consumer survey that identifies the temporal preference order for a pair of product categories. We used two beer categories, and to vary the degree of self-control the categories selected were ordered substitutes within the pair (alcoholic beer vs. non-alcoholic beer). To test the pairwise temporal preference ordering between the categories, the pair and the categories within it were presented to the subject sample. The participants rated the pair (on a nine-point scale anchored at one and nine for the two categories, with the midpoint being indifference) based on the category that they would prefer to consume. Initially they were instructed to consider only the immediate consequences (hereafter, i) of consumption (e.g., taste, fun, temptation, social perception, or any other short-term benefit) and assume identical delayed consequences (e.g., long-term social or health effects or any other long-term costs or benefits). Then they made another choice based on delayed consequences (hereafter, d) of consumption, assuming identical immediate consequences (see questions Q5–6 of our questionnaire).

The next step was to sort responses depending on whether the individual preferred one category from one temporal perspective, but expressed the reverse preference from the other temporal perspective (d > 0 and i < 0, or d < 0 and i > 0), or whether the individual (at least weakly) preferred the same category in a pair from both temporal perspectives (d ≥ 0 and i ≥ 0, or d ≤ 0 and i ≤ 0). Positive temporal reverse scores (d−i > 0, because d > 0 and i < 0) mean that the category was preferred based on the delayed consequences but not considering immediate consequences; thus, it is seen as a relative virtue while the other category in the pair is a relative vice. (For example (adapted from [1]), if an individual marks 1 on the immediate scale (indicating a preference for one anchor; e.g., alcoholic beer) and 8 on the delayed scale (indicating a preference for the other anchor; e.g., non-alcoholic beer), these values are rescaled to −4 (immediate) and +3 (delayed). The temporal reversal score (delayed minus immediate) would be +7, so that alcoholic beer would be classified as a relative vice and non-alcoholic beer as a relative virtue). In addition, negative temporal reverse scores (d−i < 0, because d < 0 and i > 0) mean that the category not preferred based on the delayed consequences was preferred from the immediate consequences, making it a relative vice while the other category in the pair is a relative virtue. Finally, a pair in which a category was (at least weakly) preferred in both temporal perspectives—immediate and delayed (d ≥ 0 and i ≥ 0, or d ≤ 0 and i ≤ 0)—received a temporal reversal score of zero (d−i = 0), which indicates no inconsistent temporal preferences. This latter case would represent a product with delayed reward (light or diet product) and no immediate cost (d ≥ 0 and i ≥ 0), or a product with immediate reward and no delayed cost (regular product) (d ≤ 0 and i ≤ 0), respectively. In virtue of this classification criteria, and given that in Spain alcoholic beer of low alcoholic strength is perceived as a natural product that forms part of a healthy Mediterranean diet [10], we can expect that alcoholic beer would be identified as a regular product with no delayed cost (it does not induce time-inconsistent preferences), and thus alcoholic beer could not be classified as a relative vice or virtue product according to time-inconsistent preferences.

### 3.2. Threshold of Consumption at Which Virtue Turns into Vice

Assuming that the pair of beer products cannot be characterized as a relative vice nor relative virtue, and if alcoholic beer is considered a regular product with no delayed cost (with average values of d ≤ 0 and i ≤ 0), we tested whether there is a certain consumption threshold of alcoholic beer at which it turns from a virtue into a vice product. In order to establish the threshold, we analysed the health effects of moderate beer consumption in Spain. This review led to the consideration that, from a health point of view, moderate beer consumption in Spain has been established as 500 mL per day [39]. Given that half a litre per day is considered to be the quantity that is the recommended health guideline in Spain [10], and that [40] conducted an experiment that considered two cans of beer as corresponding to a moderate consumption of alcohol in Spain, we determine three cans of alcoholic beer as a large amount and one can as a small amount.

The subjects used (see questions Q10–11 of our questionnaire) a seven-point scale to evaluate the future consequences of drinking one can and of drinking three cans (1 = negative consequences and 7 = positive consequences) of a new alcoholic beer. Then, we examined the difference between the delayed consequences of consuming large amounts (three cans) and small amounts (one can) of alcoholic beer. Thus, we determined whether small amounts of alcoholic beer were perceived to confer long-term health benefits while large quantities were considered to impose a delayed cost.

### 3.3. Analysis of the Lack of Consumption Self-Control through an Experiment Involving the Effect of Price Promotions on Purchase Quantity

The evidence regarding a threshold of consumption at which virtue turns into vice (in the context of the alcoholic beer with no delayed cost) would suggest that this product does not impose self-control over consumption, which could lead to a situation in which the quantity purchased is influenced exclusively by price promotion, rather than whether the beer is alcoholic. We analysed this implication through the effect of quantity discounts on purchases of beer and on rationing of purchases of beer; in other words, we examined the moderating role of alcoholic/non-alcoholic beer in the effect of quantity discounts on the amount purchased.

To test whether consumers of alcoholic beer are more or less price sensitive than consumers of non-alcoholic beer, we used the methodology applied by [1], which examines consumer demand in response to two different quantity discounts offered for the same purchase quantity. The experiment applied manipulated inconsistency conditions of the inter-temporal preferences and the potential need for self-control, describing the beer as 5% alcohol (vice product) or 95% alcohol free (virtue product) (Frame factor).

Specifically, the subjects had the opportunity to buy zero, one, or three cans of a new brand of beer at different prices. They were previously informed that 10% of the participants would be selected at random to receive €10, and the winners would have to buy the number of cans they had chosen of a well-known brand of beer at the price given in the questionnaire. This procedure prevented individuals from undervaluing their true demand at a given price as they would be losing the chance to buy more preferable beer at the same price. Similarly, individuals that overvalued their true demand at a given price would have to buy beer that they would rather not buy at that price.

The experiment used a 2 × 2 factorial-type design in which the new brand of beer was described as having 5% alcohol to one group and as being 95% alcohol free to the other group (Frame). This manipulation, adopted by [41] and [1], makes the inter-temporal consequences of consumption vary while the information stimulus is considered fixed. Thus, beer with 5% alcohol was expected to be preferable when only the immediate consequences were considered, whereas beer that is 95% alcohol free was anticipated to be preferred when only the delayed consequences were considered. The other factor was the depth of the quantity discount offered (see question Q7 of our questionnaire). All participants were offered the choice of a small purchase quantity of one can of beer for €1 or a large quantity of three cans. One group was offered a small discount of three cans for €2.80, and the other group was offered a large discount of three cans for €1.80. As manipulation controls, the participants used a seven-point scale to evaluate their perception of the taste of the new brand after drinking one can and after drinking three cans (1 = bad taste and 7 = good taste); the future consequences of drinking one can and of drinking three cans (1 = negative consequences and 7 = positive consequences); and the price of three cans in relation to the price of one can (1 = not expensive and 7 = expensive) (see questions Q8–12 of our questionnaire).

A logistic regression analysis was applied to predict the purchase quantity probabilities for the individuals that purchased one or three cans of beer. The dependent variable was a dummy that took a value of 1 if one can was chosen and 0 if three cans were chosen. To detect rationing effects, non-buyers (44 students) were excluded from the analysis of quantities bought, as we were unable to distinguish whether these subjects were exercising self-control or simply did not like beer. The independent variables are: (i) the variable dummy of the frame, with a value of 1 for the 95% alcohol-free frame, and 0 for the 5% alcohol frame; and (ii) the dummy variable of the discount, with a value of 1 for the small discount, and 0 for the large discount. In the context of an alcoholic beer with no delayed cost, we expected that the purchased quantity would be influenced exclusively by price promotion, rather than whether the beer was alcoholic; this would suggest that it does not impose self-control.

In any case, the alcoholic–non-alcoholic beer manipulation of the above experiment only set conditions for impulsive behaviour, but did not directly measure the resulting need for self-control. In other words, the purchasers of alcoholic beer could have restricted the amounts bought not to control the temptation to consume, but because they prefer to consume at lower prices as consuming at higher prices brings more negative delayed consequences. Consuming at lower prices allows buyers of vice products to enjoy the taste without having to worry about these delayed consequences [1]. To solve this inconvenience in the above experiment and to find out whether the results obtained were due to purchase quantity rationing rather than to differences in consumption rates, we included a measure of the need for self-control through the participants’ scoring on the 12-item consumer impulsiveness scale posited by [42] (see questions Q13–Q24 of our questionnaire). Consequently, if the participants used purchase quantity rationing as a mechanism of self-control, it could be expected that the hedonic individuals (high need for self-control) would ration their purchase quantities of alcoholic beer more than the prudent individuals would (lower need for self-control).

### 3.4. Analysis of the Lack of Reverse Self-Control through an Experiment Involving the Effect of Passage of Time on Anticipated Regret of Choice of Alcoholic vs. Non-Alcoholic Beer Purchases

Evidence of a threshold at which virtue turns into vice (in the context of the alcoholic beer with no delayed cost) would lead us to criticize the definition of relative vice and virtue products according to time-inconsistent preferences by suggesting that it does not impose reverse self-control. In order to examine the reverse self-control assumption, we analysed whether a greater temporal separation between a choice and its assessment increases the regret of virtuous decisions on beer category. To this end, we adapted an experiment by [22] to the case of alcoholic and non-alcoholic beer (see questions Q1–4 of our questionnaire). A self-control dilemma was described, as the entire sample (N = 176) had to choose between two beers: a tasty, alcoholic beer and a tasteless, non-alcoholic beer. The students were asked to indicate the degree of regret they would feel the day after choosing either of the two beers using a scale of 1 = low anticipated regret to 7 = high anticipated regret. The students also stated their anticipated degree of regret in 10 years’ time after choosing either of the two beer categories. Thus, we were able to identify any reverse self-control used in the consumption (hyperopia) of beer if, with the passage of time, choices of virtue over vice evoked increasing regret for alcoholic beer.

## 4. Results

### 4.1. Identification of Specific ‘Vice’ Products That Do Not Impose a Delayed Non-Pecuniary Cost

Following [1]’s procedure, we calculated the mean temporal reversal score for the pair of beers across all subjects (see Table 1).

The results reveal that consumers preferred the same category from both temporal perspectives because alcoholic beer was at least weakly preferred from immediate and delayed perspectives (d ≤ 0 and i ≤ 0); thus, this pair of beer products did not induce time-inconsistent preferences and they cannot be characterized as either relative vice or relative virtue products. The pair of beer products presents average values of d that are not statistically different from zero, but those of i are negative and significantly different from zero, while mean temporal reverse scores differ from zero at a 99% confidence level. Thus, the alcoholic beer gives an immediate reward ($\bar{i}$ = −1.59 < 0; *p* < 0.0000), but its delayed reward is not statistically significant ($\bar{d}$ = −0.06 = 0; *p* > 0.10). That is, there is no evidence that alcoholic beer is a vice product that imposes a delayed cost so it can be identified as a regular product with no delayed cost.

### 4.2. Threshold of Consumption at Which Virtue Turns into Vice

The evidence obtained in Section 4.1 (that alcoholic beer is a regular product with no delayed costs) allows us to expect that there is a threshold of alcoholic beer consumption (three cans) at which this virtue product turns into a vice product.

The tests for differences between means of our student sample reveal that small amounts of alcoholic beer (one can) were perceived to confer long-term health benefits (mean = 4.37), while large quantities (three cans) were considered to impose a delayed cost (mean = 3.51), with the difference between them being statistically significant (F = 8.31, *p* = 0.005). This result shows that three cans of beer is the threshold at which this virtue product becomes a vice product.

### 4.3. Analysis of the Lack of Consumption Self-Control through an Experiment Involving the Effect of Price Promotions on Quantity Purchases

The evidence of a consumption threshold for alcoholic beer at which virtue turns into vice (see Section 4.2) leads us to expect that self-control will not manifest in the pair of beers, as defined according to time-inconsistent preferences. We thus performed an experiment to predict the purchase quantity probabilities for the participants that purchased one or three cans of beer (alcoholic beer—vice vs. non-alcoholic beer—virtue) through a logistic regression analysis.

The logistic regression analysis (see Table 2) predicted the purchase quantity probabilities for one or three cans of beer. The lack of statistically significant differences (see Table 3) between the negative consequences of consuming alcoholic and non-alcoholic beer (manipulation checks) at the moment of purchase is reflected in the quantity purchased (see Table 2). In fact, the results in Table 2 show that only the coefficient of the variable quantity discount (β_discount_ = 1.92) is statistically significant (as demonstrated by the Wald test with a coefficient above 4: χ^2^ = 12.72) and has a positive sign. This suggests that consumers preferred a large package size when there was a larger quantity discount as opposed to a smaller discount; in the same way, they preferred a small package size when there was a smaller quantity discount (e.g., if the discount was small, the probability of buying small amounts was multiplied by 6.79). The coefficient of the frame variable (vice and virtue product) is not significant (β_frame_ = 0.57, *p* = 0.285; χ^2^ = 1.14). In addition, also not significant is the coefficient of the interaction between frame and discount (β_frame*discount_ = −0.21, *p* = 0.813; χ^2^ = 0.06) on the amount purchased. In brief, it seems that purchased quantity is associated exclusively with price promotion (and that the quantities purchased are not dependent on the vice or virtue nature of the product), due to the lack of consumption self-control obtained. [To rule out a possible effect of gender in the analysis made in Table 2, a mean difference test (t = 0.13, *p* = 0.893) was performed, which revealed that there are no statistically significant differences in the amount of cans purchased for gender reasons].

Similar results were obtained when the above model included purchased quantity rationing in the form of a consumer impulsiveness scale (hedonism variable). The results (see Table 2) continue to show a positive and statistically significant coefficient for the variable quantity discount. However, the coefficient of the hedonism variable (1 = prudent consumer and 0 = hedonistic consumer) is not significant. The coefficient of the interaction between frame, discount, and hedonism on purchase quantity is not significant either. In consequence, we can say that purchase quantity is only associated with price promotion.

#### Manipulation Checks of the Experiment

With regard to the manipulation checks of the experiment, Table 3 shows the average values of the control variables. The variance analysis shows that, as expected, the participants perceived three cans of beer with a small discount (mean = 4.38) as more expensive than with a large discount (mean = 2.76) (F = 47.70, *p* = 0.000). In addition, in terms of delayed consequences, the results logically show that delayed consequences were rated better for drinking one can (mean = 4.13) than for drinking three cans (mean = 3.68; F = 6.05, *p* = 0.014) for both the vice and virtue frames. However, more detailed analysis of the control variables for the frames of vice and virtue product shows unexpected results according to the self-control argument cited by [1]. That is, no statistically significant differences were found in the negative consequences of consuming beer in the vice and virtue frames for either of the quantities consumed. Thus, we found no differences in the negative consequences of consuming one can of alcoholic beer (mean = 4.37) and one can of non-alcoholic beer (mean = 3.98; F = 2.17, *p* = 0.143), or between consuming three cans representing a vice (mean = 3.51) and three cans representing a virtue (mean = 3.78; F = 0.924, *p* = 0.338). In other words, at the moment of purchase, the participants considered that consuming alcoholic beer (5% alcohol) had similar negative consequences to those of non-alcoholic beer (95% alcohol-free) and, therefore, that the delayed consequences of alcoholic beer were no worse than those of non-alcoholic beer. Thus, the consumers were indifferent to alcoholic beer from the delayed consequences perspective, and a lack of self-control was evident.

With regard to the immediate consequences, the results show that taste presents significant differences between three cans (mean = 4.40) and one can (mean = 3.86) for both the vice and virtue frames (F = 7.92, *p* = 0.005). Consuming one can of alcoholic beer was perceived as having better immediate consequences (taste) (mean = 4.46) than one can of non-alcoholic beer (mean = 3.49; F = 12.44, *p* = 0.001), and consuming three cans of alcoholic beer was perceived to have a better taste (mean = 4.68) than three cans of non-alcoholic beer (mean = 4.22; F = 2.89, *p* = 0.091).

### 4.4. Analysis of the Lack of Reverse Self-Control through an Experiment Involving the Effect of Passage of Time on Anticipated Regret of Choice of Alcoholic vs. Non-Alcoholic Beer Purchases

The evidence found in Section 4.2 (that there is a threshold at which alcoholic beer consumption becomes vice) leads us to expect that reverse self-control will not manifest in the pair of categories of beer defined according to time-inconsistent preferences. We tested whether a greater temporal separation between a choice and its assessment enhances regret regarding a virtuous decision given that excessive control and farsightedness (hyperopia) can have negative long-term consequences.

**Figure 1:**
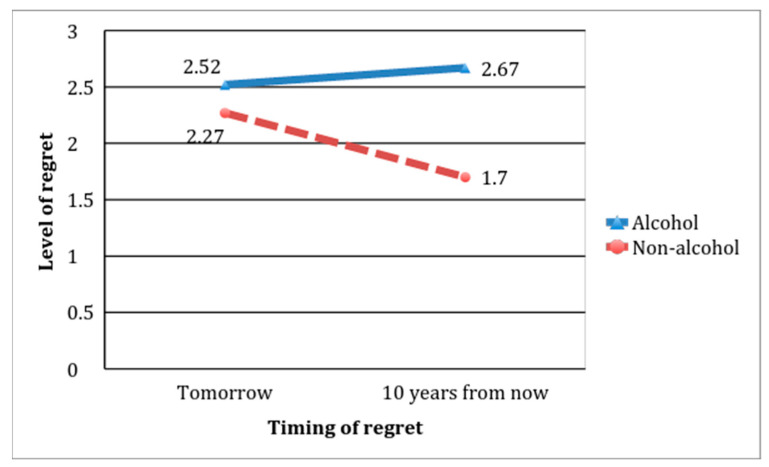
Regret when choosing non-alcoholic beer vs. alcoholic beer.

The tests for differences between means of our student sample (see Figure 1) do not show that a longer temporal perspective (tomorrow versus in 10 years’ time) led to an increase in anticipated regret. In fact, the opposite was found: a longer temporal perspective led to a reduction in anticipated regret for choosing the non-alcoholic beer relative to the anticipated regret of choosing the alcoholic beer. Thus, the interaction between anticipated regret and temporal perspective is significant (F = 10.13, *p* = 0.000); and, as expected, neither the main effect of regret nor of temporal perspective has statistical significance. This means that a longer temporal perspective reduced the anticipated regret from choosing non-alcoholic beer (mean = 1.73 in the distant future versus mean = 2.27 in the near future for anticipated regret; t = 3.25, *p* = 0.001). However, a longer temporal perspective maintained the anticipated regret from choosing alcoholic beer (mean = 2.67 in the distant future versus mean = 2.52 in the near future for anticipated regret; t = −0.71, *p* = 0.477); thus, it seems that the characterization of alcoholic beer as a regular product with no delayed costs (see Section 4.1) attenuated the effect of temporal perspective on self-control regrets. In summary, these results do not show any reverse self-control in consumption (hyperopia) for beer and therefore, with the passage of time, choices of virtue over vice do not evoke increasing regret when it comes to alcoholic beer.

### 4.5. Discussion

The results of Section 4.1 show that the alcoholic beer can be identified as a regular product with no delayed cost, but not a vice product that imposes a delayed cost. That is, consumers preferred the same category from both temporal perspectives; thus, this pair of beer products (alcoholic beer; non-alcoholic beer) did not induce time-inconsistent preferences. This result does not support the classic definition of relative vices of [1,6], among others; and it supports the idea that the relevant question would be whether there is a certain consumption threshold at which a virtue turns into a vice.

In fact, the results of Section 4.2 find that consuming large quantities of this product imposes a delayed cost. Specifically, three cans of beer is the threshold at which this virtue product becomes a vice product. Therefore, it seems there is a fine line between healthy and risky consumption of a product, in that consuming small or moderate quantities can be perceived as healthy [32,43], and that small food portions in small packages represents the diet food version [29].

The results of logistic regression in the Section 4.3 show that the coefficient of the frame variable (vice and virtue product) is not significant on the amount purchased; while the coefficient of the quantity discount has an influence on the amount purchased. It seems that self-control does not manifest in the pair of beer, as defined according to time-inconsistent preferences assumption of [1].

The results of Section 4.4 evidence that a longer temporal perspective does not lead to a reduction in anticipated regret for choosing the non-alcoholic beer relative to the anticipated regret of choosing the alcoholic beer. It seems that reverse self-control (excessive control and farsightedness; hyperopia), assumed by [22] and [23], does not manifest in the pair of categories of beer defined according to time-inconsistent preferences.

All these results do not support the definition of relative vices according to time-inconsistent preferences. They allow us to identify alcoholic beer as a regular product that does not impose a delayed non-pecuniary cost, by revealing a threshold at which a virtue turns into a vice. Thus, our results would support the idea of [13] that establishing the healthiness of a product depends on the quantity eaten.

Finally, the results of our study can also contribute to the debate in many firms [44] around whether to continue offering unhealthy products while announcing plans to improve nutrition. There is room for doubt on this, given the lack of agreement on the definition of ‘healthy’ and ‘unhealthy’ food:

‘A carrot is clearly healthy and a sweet fizzy drink is not, but the distinction is not always as obvious as that. A company may reduce the sugar content of a biscuit, but that does not make it healthy. A hamburger may be ‘energy dense’, as nutritionists put it, with a lot of calories packed in, but it has some nutritional value. Even a deep-fried Oreo, a cannonball of fat and sugar, will not doom the consumer to obesity if eaten only occasionally’ [33].

An important implication of a definition focused on the consumption threshold is that companies should intensify the use of nutritional labelling in their food products to indicate the amount of a nutrient that a food provides relative to the recommended amount [20,26]. In the same way, health educators and policy makers, instead of using the common refrain ‘there is no such thing as good and bad foods, only good and bad diets’ to define specific foods as unhealthy, should provide consumers with practical tools to facilitate healthy dietary choices, such as the nutrient profile of a food product both in absolute terms and in relation to other food products [27].

## 5. Conclusions

This study aimed to provide evidence that specific ‘vice’ products do not always impose a delayed non-pecuniary cost and that consuming such products in large quantities imposes a delayed cost. In addition, by applying two experiments, we estimated the potential self-control in relation to consumption and reverse self-control of consumption.

Our analysis of the survey data reveals that alcoholic beer does not impose a delayed non-pecuniary cost. In addition, our empirical analysis of the data shows a threshold for alcoholic beer at which it turns from a virtue product into a vice product, which may explain the lack of consumption self-control and reverse consumption self-control demonstrated. This threshold may be justified in health sciences because consuming small or moderate amounts of alcohol is healthy [30,31]; and in consumer research because small food quantities in small packages represent the ‘diet’ version of the product [28].

The management implications of these results are as follows. The findings of this study provide valuable information on whether decision making should focus on promotions or on consumer choice regarding the consumption quantity. Basically, the larger effect of price promotions on virtue over vice purchase quantity, as detected by [1], suggests that vice consumption self-control could lead marketing managers to segment and differentiate prices, offering a variety of packet sizes that in particular include small-sized vice products with price premiums, as opposed to the discounts applied to virtue products. However, the lack of consumption self-control and reverse consumption self-control obtained in our study for alcoholic beer make this price segmentation and differentiation unnecessary. In fact, dietary-restrained consumers do not view smaller packaged snacks as portion control devices [45]. In consequence, the priority is to distinguish vice and virtue products (according to time-inconsistent preferences) from regular products with no delayed cost, and from light or diet products with delayed reward and no immediate cost (products that do not induce time-inconsistent preferences). In addition, the result that the quantity purchased was influenced by price promotion, rather than whether the beer was alcoholic, allows us to suggest that the relevant question is whether consumption above a certain threshold turns a virtue into a vice product.

As with other studies, this paper has certain limitations. First, our analysis of beer categories impedes a generalization of the results and deep understanding of the behaviour regarding choice of products whose negative consequences of consumption depend on the quantity consumed. Despite this, some studies, such as [46], have detected that other products, like sugar and regular coffee, can also be classified as regular products with no delayed cost. This accumulation of similar results for different products; sugar and regular coffee (see [46]), as well as alcoholic beer (in our paper), suggests that the definition of relative vice and virtue can be criticized because people may consume sugar, regular coffee and alcoholic beer that provide an immediate reward while not regarding these products as imposing a delayed non-pecuniary cost. Thus, this evidence of the existence of regular products with no delayed cost for different product categories, supports the insights about the existence of a consumption threshold after which virtue products become vice products. Another limitation is the absence of data on consumer perceptions. Interesting research threads that could be developed in the future could, first, include data on other products that do not have a delayed cost, which would allow for corroboration of our findings; and second, reveal the motivations behind the purchase quantity choices observed.

## Figures and Tables

**Table 1:** Product categories.

Product Category	Immediate Consequences *I*	Delayed Consequences *D*	Product Category	Mean Temporal Reversal Score	N
Alcoholic beer	−1.590 *****	−0.056	Non-alcoholic beer	1.01 *****	176

***** *p* < 0.0001 in two-sided test.

**Table 2:** Effect of the frame and of discount on purchase quantities (standard error in brackets).

Variable	Coefficient	Coefficient
Intercept	−1.897 ** (0.619)	−1.609 * (0.775)
Frame	0.569 (0.533)	0.606 (0.478)
Discount	1.915 *** (0.537)	1.946 **** (0.482)
Frame * discount	−0.212 (0.895)	
Hedonic		−0.132 (0.440)
Frame * discount * hedonic		−0.466 (0.992)
χ^2^ (−2 log likelihood)	20.603	20.963
Degree of freedom	3	4
N	132	132

* *p* < 0.05, ** *p* < 0.01, *** *p* < 0.001, **** *p* < 0.0001.

**Table 3:** Average values of the control variables (N = 176).

	Vice Frame		Virtue Frame		
Immediate and delayed consequences according to quantity	Small Discount	Large Discount	Small Discount	Large Discount	F
Delayed consequences after drinking one can	4.43	4.27	4.20	3.85	1.116
Delayed consequences after drinking three cans	3.57	3.42	3.95	3.68	0.543
Immediate consequences: Taste after drinking one can	4.24	4.81	3.80	3.31	5.399 ***
Immediate consequences: Taste after drinking three cans	4.90	4.31	4.00	4.35	1.969
Three cans is expensive relative to one can	4.57	2.88	4.18	2.71	16.388 ****

*** *p* < 0.001, **** *p* < 0.0001.

## Data Availability

Not applicable.

## Appendix A. Questionnaire

### Appendix A.1. Regret in the Choice of Beer Type

Q1–2. If faced with the choice of two different types of beer; a delicious alcoholic beer or a healthy non-alcoholic beer. Imagine you have just chosen the alcohol free beer, indicate the level of regret you would anticipate as feeling tomorrow for having chosen non-alcoholic beer.

Q1. Low anticipated level of regret tomorrow for choosing non-alcoholic beer today	1	2	3	4	5	6	7	High anticipated level of regret tomorrow for choosing non-alcoholic beer today

Imagine you have just chosen the alcoholic beer, indicate the level of regret you would anticipate as feeling tomorrow for having chosen alcoholic beer today.

Q2. Low anticipated level of regret tomorrow for choosing alcoholic beer today	1	2	3	4	5	6	7	High anticipated level of regret tomorrow for choosing alcoholic beer today

Q3–4. Indicate the level of regret you would anticipate as feeling in ten years’ time for having chosen non-alcoholic beer today.

And the level of regret you would anticipate as feeling in ten years’ time for having chosen alcoholic beer today.

Q3. Low anticipated level of regret in ten years for choosing non-alcoholic beer today	1	2	3	4	5	6	7	High anticipated level of regret in ten years for choosing non-alcoholic beer today
Q4. Low anticipated level of regret in ten years for choosing alcoholic beer today	1	2	3	4	5	6	7	High anticipated level of regret in ten years for choosing alcoholic beer today

### Appendix A.2. Consequences of Consumption of Type of Beer

Q5. If you were a consumer of both of the two following types of beer and their consumption had identical long term consequences (e.g.: long-term health or social effects or any other cost or benefit in the long term), which of the two types of beer would you consume considering only the short term effects? To evaluate these short term effects, think of flavour, fun, fashion, temptation or any other factor that could make the consumption of these products agreeable. (1: “Extremely prefer alcoholic beer”; 9: “Extremely prefer non-alcoholic beer”)

Q5. Alcoholic beer	1	2	3	4	5	6	7	8	9	Non-alcoholic beer

Q6. If you were a consumer of both the two following types of beer and their consumption had identical short term consequences (such as flavour, fun, fashion, temptation or any other factor that could affect the degree of enjoyment in the short term), which of the two types of beer would you consume considering only the long term effects? To evaluate long term effects think of social or health consequences. (1: “Extremely prefer alcoholic beer”; 9: “Extremely prefer non-alcoholic beer”)

Q6. Alcoholic beer	1	2	3	4	5	6	7	8	9	Non-alcoholic beer

### Appendix A.3. Purchase/Consumption Quantity of Beer Type

Q7a. Using as a reference a 33cL can of a new brand of beer with 5% alcohol, imagine you have the opportunity to buy 1 can for 1 € or 3 cans for 2.80 €. How many cans would you buy when presented with this offer?

	**Purchase Choice**		
Beer	0 cans	1 can for 1 €	3 cans for 2.80 €
Q7a. 5% alcohol beer			

Q8–9a. Indicate how you would perceive the flavour of the new brand of beer with 5% alcohol after one can, and after 3 cans.

Q8a. Bad flavour after one can	1	2	3	4	5	6	7	Good flavour after one can
Q9a. Bad flavour after three cans	1	2	3	4	5	6	7	Good flavour after three cans

Q10–11a. Indicate how you would perceive the future consequences of drinking; one can, and 3 cans of the new beer brand with 5% alcohol.

Q10a. Bad future consequences after 1 can	1	2	3	4	5	6	7	Good future consequences after 1 can
Q11a. Bad future consequences after 3 can	1	2	3	4	5	6	7	Good future consequences after 3 cans

Q12a. How would you evaluate the price of 3 cans in relation to the price of 1 can of this new beer brand with 5% alcohol (see question 7)?

Q12a. Cheap	1	2	3	4	5	6	7	Expensive

### Appendix A.4. Type of Beer Consumer

Read the following adjectives carefully and indicate how they would describe you. Circle one number on the scale for each adjective, where 1 indicates the adjective could be used to describe you usually, 4 indicates it could be used to describe you sometimes and 7 indicates that it would never be used to describe you.

	**Describes Me Usually**			**Describes Me Sometimes**			**Never Describes Me**
Q13. Impulsive	1	2	3	4	5	6	7
Q14. Careless	1	2	3	4	5	6	7
Q15. Self-controlled	1	2	3	4	5	6	7
Q16. Extravagant	1	2	3	4	5	6	7
Q17. Farsighted	1	2	3	4	5	6	7
Q18. Responsible	1	2	3	4	5	6	7
Q19. Restrained	1	2	3	4	5	6	7
Q20. Easily tempted	1	2	3	4	5	6	7
Q21. Rational	1	2	3	4	5	6	7
Q22. Methodical	1	2	3	4	5	6	7
Q23. Enjoy spending	1	2	3	4	5	6	7
Q24. A planner	1	2	3	4	5	6	7

Four different questionnaire types were posed that changed the formulation of the questions Q7 to Q12 as follows:

Questionnaire type “b”:

Q7b. Using as a reference a 33cl can of a new brand of beer with 5% alcohol, imagine that you have the opportunity to buy one can for 1 € or three cans for 1.80 €. How many cans would you buy when presented with this offer?

Presented similar to questions Q8b to Q12b.

Questionnaire type “c”:

Q7c. Using as a reference a 33cl can of a new brand of beer 95% alcohol free, imagine that you have the opportunity to buy one can for 1 € or three cans for 2.80 €. How many cans would you buy when presented with this offer?

Presented similar to questions Q8c to Q12c.

Questionnaire type “d”:

Q7c. Using as a reference a 33cl can of a new brand of beer 95% alcohol free, imagine that you have the opportunity to buy one can for 1 € or three cans for 1.80 €. How many cans would you buy when presented with this offer?

Presented similar to questions Q8d to Q12d.
